# Apparent effect of rabbit endogenous lentivirus type K acquisition on retrovirus restriction by lagomorph Trim5αs

**DOI:** 10.1098/rstb.2012.0498

**Published:** 2013-09-19

**Authors:** Melvyn W. Yap, Jonathan P. Stoye

**Affiliations:** Division of Virology, MRC National Institute for Medical Research, The Ridgeway, Mill Hill, London NW7 1AA, UK

**Keywords:** endogenous retroviruses, restriction, evolution

## Abstract

To test the hypothesis that rabbit endogenous lentivirus type K (RELIK) could play a role in shaping the evolution of TRIM5α, the susceptibility of viruses containing the RELIK capsid (CA) to TRIM5 restriction was evaluated. RELIK CA-containing viruses were susceptible to the TRIM5αs from Old World monkeys but were unaffected by most ape or New World monkey factors. TRIM5αs from various lagomorph species were also isolated and tested for anti-retroviral activity. The TRIM5αs from both cottontail rabbit and pika restrict a range of retroviruses, including HIV-1, HIV-2, FIV, EIAV and N-MLV. TRIM5αs from the European and cottontail rabbit, which have previously been found to contain RELIK, also restricted RELIK CA-containing viruses, whereas a weaker restriction was observed with chimeric TRIM5α containing the B30.2 domain from the pika, which lacks RELIK. Taken together, these results could suggest that the pika had not been exposed to exogenous RELIK and that endogenized RELIK might exert a selective pressure on lagomorph TRIM5α.

## 1. Introduction

Retroviruses have a unique replication strategy that involves an obligatory integration step where the viral genome is inserted into that of the host. When this takes place in germ or embryonic cells, the virus can be transmitted vertically to the next generation and is termed an endogenous retrovirus (ERV). ERVs can be fixed in a population, where with time, most of them accumulate deletions, mis-sense and non-sense mutations, leading to a loss of replication competency. While some of the ERVs are co-opted by the host for various functions ranging from development to intrinsic immunity, many are just relics left behind from an ancient infection [1,2]. Like the fossils in paleontology, ERVs provide a means to estimate the age of an infection [3], which could provide insights into the origins of related current exogenous viruses that are in circulation [4,5].

Lentiviruses belong to the genus of retrovirus with members that are a threat to human health, such as HIV-1 and HIV-2. They were thought to be a group of relatively modern viruses until the discovery of the rabbit endogenous lentivirus type K (RELIK) [6]. Compared with the primate lentiviruses, RELIK has a simpler genome, containing only three accessory genes (tat, rev and dUTPase). However, similar to the primate lentiviruses, their capsid (CA) protein binds cyclophilin A [7]. In addition, viruses containing the RELIK CA can also infect non-dividing cells, suggesting that these properties of lentiviruses were acquired a long time ago. Through PCR, RELIK sequences were identified in the genomes of several genera of the lagomorph order, including that of the hare, which placed the origins of RELIK at more than 12 Mya [8]. Analyses of the PCR products revealed a high degree of homology with the original sequences isolated from the European rabbit. This suggested that invasion of the germline occurred before the divergence of the *Lepus* genus, or that the exogenous virus was widespread among the different lagomorph genera that shared common geographical distributions. However, RELIK does not seem to be present in the pika family of lagomorphs [8,9].

Lentiviruses and other retroviruses are susceptible to host restriction factors [10]. One group of restriction factors targets the CA of the virus [1]. These include the murine factor Fv1 [11], as well as TRIM5α [12] which has been found in many species [13–16]. TRIM5α belongs to a large family of proteins containing the tripartite ring, B-box and coiled coil (RBCC) motif [17,18]. Some Trim proteins such as TRIM5α also contain the B30.2 domain at the C-terminus [19,20]. TRIM5α restricts a range of retroviruses, including lentiviruses [21,22], gammaretroviruses [23], spumaviruses [24] and betaretroviruses [25]. While its precise mechanism of action is still not known, it is clear that multimerization of the molecule that is mediated by the coiled coil is essential [26–29]. The B30.2 domain with its four variable regions [30] has been found to be the major determinant of restriction specificity [31]. It mediates binding to HIV-1 CA [26] and is under strong positive selection [32,33], indicating that it is the part of TRIM5α that recognizes the virus. Presumably, TRIM5α evolution is driven by exposure to retroviral CA. However, it remains unclear whether the selective pressure comes from endogenous or exogenous viruses [1].

TRIM5α orthologues have been isolated from lagomorphs such as the European rabbit and hare [15,34]. They restrict a range of retroviruses, including gammaretroviruses and lentiviruses. In addition, there is strong evidence of positive selection in residues in the B30.2 domain, suggesting that retroviruses could be involved in shaping the evolution of the lagomorph TRIM5αs [35]. In the light of the prevalence of RELIK sequences in many lagomorph genera, we wondered whether this ancient lentivirus could play a role in the selection of the lagomorph TRIM5αs. Hence, we set out to investigate the relationship between lagomorph TRIM5α restriction and viruses containing the RELIK CA. We report here that the TRIM5α from the cottontail rabbit and pika can restrict a wide range of retroviruses. In addition, viruses containing the RELIK CA were susceptible to restriction by TRIM5αs from a number of primates and lagomorphs.

## 2. Material and methods

### (a) DNA constructs

**Figure 1.**
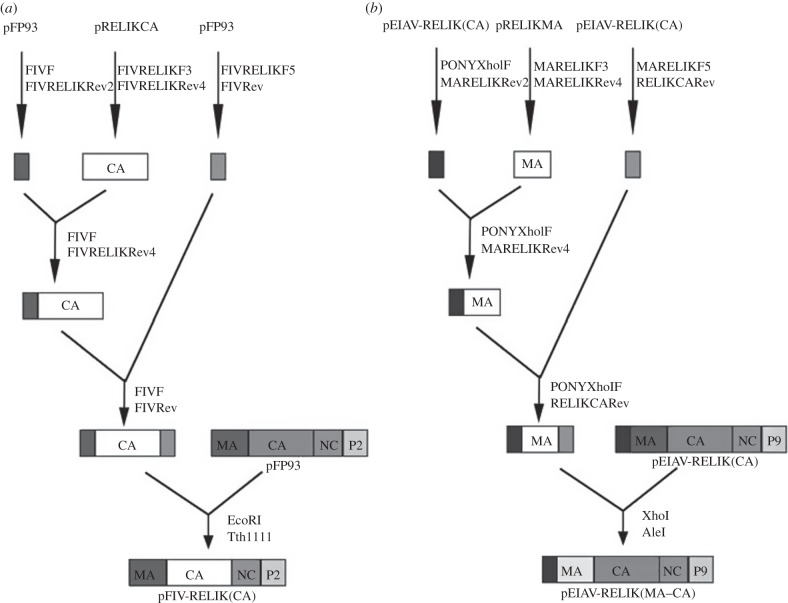
Construction of chimeric *gag* genes. Schematic of chimeric *gag* genes used. (*a*) pFIV-RELIK(CA); (*b*) pEIAV-RELIK(MA–CA).

Structures of chimeric *gag* constructs are illustrated in figure 1. The construction of pEIAV-RELIK(CA) has been described previously [7]. pFIV-RELIK(CA) was made by substituting the *Eco*RI/Tth111I fragment containing the FIV CA flanked by MA and NC sequences in pFP93 with a corresponding fragment containing the RELIK CA flanked by FIV sequences. The fragment was generated by overlapping PCR using primer pairs FIVF/FIVRELIKRev2, FIVRELIKF3/FIVRELIKRev4 and FIVRELIKF5/FIVRev in reactions with templates pFP93 [36], pRELIKCA [7] and pFP93, respectively. The first two fragments were joined in a second reaction with primer pair FIVF/FIVRELIKRev4. The resulting product was joined to the third fragment from the first reaction using primer pair FIVF and FIVRev. In order to make EIAV-RELIK(MA–CA), the *Xho*I/*Ale*I fragment from pEIAV-RELIK(CA) that contain the EIAV MA was replaced by a fragment containing MA from RELIK (synthesized by Genescript based on the sequence reported by Katzourakis *et al*. [6]). This fragment was generated using overlapping PCR by performing the first reactions with primer pairs PONYXhoIF/MARELIKRev2, MARELIKF3/MARELIKRev4 and MARELIKF5/RELIKCARev using pEIAV-RELIK(CA) [7], pRELIKMA and pEIAV-RELIK(CA) as templates, respectively. The first two fragments were joined using primer pair PONYXhoIF/MaRELIKRev4, and the resulting product was joined to the third fragment in the first reaction using primer pair PONYXhoIF/RELIKCARev.

TRIM5αs from the European (*Oryctolagus cuniculus*) and cottontail (*Sylvilagus floridanus*) rabbit were amplified from cDNAs prepared from SIRC and Sf1ep cells, respectively, using the primer pairs RabbitT5F and RabbitT5Rev. mRNAs from these cell lines were isolated using the total mRNA extraction kit from Qiagen and reverse transcribed to cDNA using the first strand synthesis kit from Roche. The TRIM5α PCR products were cloned into pENTR-D-TOPO (Invitrogen) and sequenced. They were then transferred into pLGatewayIEYFP and pLGatewaySN by an LR recombination reaction using LR Clonase to create the retroviral delivery vectors.

A draft genomic DNA sequence from American pika (*Ochotona princeps*) can be found at http://www.ensembl.org/Ochotona_princeps/Info/Index/. From this sequence, primers targeting sequences in TRIM5α exon7 and exon8 were designed and used to amplify the B30.2 region from pika genomic DNA (a generous gift of Dennis Lanning, Loyola University). The product was cloned into TopoBlunt and sequenced. Nucleotide and predicted amino acid sequences of the B30.2 coding region were identical to that in the database. To test the restriction properties of pika TRIM5α, the B30.2 domain was fused to the RBCC of the TRIM5α from the European rabbit. This was achieved by overlapping PCR using the primer pair TopoRabbitT5F/SIRCPIKARev3 to amplify the sequences encoding the RBCC from the European rabbit TRIM5α and primer pair SIRCPIKAF3/PIKARev to amplify exon8 from the Pika TRIM5α encoding the B30.2 domain. The two PCR products were used in a second reaction with primer pair TopoRabbitT5F/PIKARev to generate the chimeric TRIM5α that was cloned into pENTR/D-Topo before transferring to pLgatewayIRESEYFP and pLgatewaySN by LR recombination. Sequences of these primers are shown in table 1.

**Table 1.** Oligonucleotide sequences.

primer name	primer sequence
FIVF	TTTTAAATATGACGGTGTCTACTG
FIVRELIKRev2	ATTCCTGACGTCCATTTACTGTTTGAATAGG
FIVRELIKF3	AGTAAATGGACGTCAGGAATATGAACCGGTTG
FIVRELIKRev4	GCATTTTATAGCTGGTGCTGCCAAATTCAC
FIVRELIKF5	CAGCACCAGCTATAAAATGCAACTCTTGGC
FIVRev	CCAGTTTCCCGAATTCTTTCTATTTC
PONYXhoIF	ATAGGCTAGCCTCGAGGTCGAC
MARELIKRev2	TCCCACCCATCTTACCTGTCCTCCTGTGTTC
MARELIKF3	GACAGGTAAGATGGGTGGGACGTCCCAGTC
MARELIKRev4	ATTCTTCAGATTCTTTTGCTTTTTCTTCCTTAC
MARELIKF5	AGCAAAAGAATCTGAAGAATATCCAATCATG
RELIKCARev	TGTTGATATCCACGCTGGTGAAG
RabbitT5F	CACCATGGCTTCAGCAATCTTAGCG
RabbitT5Rev	TCAACAGCTCAACTCGCAGATTG
SIRCPIKARev3	GTCACGTTGGCCCAATAGCGCTGGGCATGTC
SIRCPIKAF3	CGCTATTGGGCCAACGTGACATTGACTCCAAG
PIKARev	CTAGCAAAGCGTCATGGGTCTTG

### (*b*) Cells and virus production

SIRC (European rabbit), CrFK (cat), D17 (dog), TE671 and 293T (human) cells were maintained in DMEM containing 10% FCS and 1% antibiotics, whereas Sf1ep (cottontail rabbit) cells were maintained in MEM containing 10% FCS, 1% antibiotics and 1% non-essential amino acids. Viruses were made by transfection of 293T cells as described previously. To make the delivery viruses, pVSVG, pHIT60 and either pLgatewayIRESEYFP or pLgatewaySN carrying the various primate and lagomorph TRIM5αs was used. EIAV and the EIAV-RELIK chimeric viruses were made by transfecting pVSVG, pONY8.4ZCG [37] and either pONY3.1 [37], pEIAV-RELIK(CA) or pEIAV-RELIK(MA–CA), whereas FIV and FIV-RELIK(CA) were made by transfecting pVSVG, pFIVGFP [36] and either pFP93 or pFIV-RELIK(CA), respectively. The virus-containing medium was harvested 48 h post-transfection and concentrated 100-fold by ultracentrifugation for 2 h at 19 500 rpm in a SW28 rotor. Plasmids for producing MLV, HIV-1, HIV-2, SIVmac, PFV, SFV and FFV have been described previously [7,23,24,31,38].

### (c) Infectivity and restriction assays

The infectivities of the RELIK chimeric viruses were measured by endpoint titration. Ten-fold dilutions of the virus stocks were made and added to each well of a 12-well plate that had been seeded with 5 × 10^4^ cells per well the day before in the presence of 10 μg ml^−1^ of polybrene. The cells were then stained for β-galactosidase 2 days post-transduction, and the number of blue cells counted [39].

To study the restriction of primate TRIM5αs on EIAV and EIAV-RELIK(CA), vectors carrying the primate TRIM5αs as well as EYFP were used to deliver the genes into CrFK cells so that more than 80 per cent were transduced. Two days post-transduction, the cells were seeded into 12-well plates and challenged with different dilutions of the EIAV and EIAV-RELIK(CA) virus. The number of infected cells was determined by staining for β-galactosidase 2 days later.

The restriction phenotype of the lagomorph TRIM5αs was tested by the two-colour FACS assay as described previously. Briefly, CrFK cells were transduced with delivery vectors carrying TRIM5α and the EYFP marker. These were challenged with a panel of retroviruses (HIV-1, HIV-2, SIVmac, FIV, EIAV, PFV, SFV, FFV, B-MLV and N-MLV) 2 days post-transduction. The percentages of cells with and without restriction factor that were infected were determined by FACS analyses.

Cat CrFK cells were found to be permissive for the EIAV-RELIK chimeric virus. Single cell clones expressing lagomorph TRIM5αs were derived from CrFK cells by transduction with limiting dilutions of retroviral vectors carrying both TRIM5α as well as the G418 resistance marker. The transduced cells were selected on medium containing G418 (1 mg ml^−1^) for two weeks until visible colonies appeared. Well-separated colonies were picked, expanded and tested for restriction.

### (d) Protein analyses

Viruses were pelleted through a 20% sucrose cushion at 100 000*g* for 3 h at 4°C. The pellets were washed once with PBS and resuspended in SDS loading buffer before separation by SDS–PAGE. The protein bands were then stained with Imperial protein stain (Thermo Scientific).

### (e) Nucleotide sequence accession numbers

The gene sequences determined in this study have been submitted to GenBank (accession nos KC425460 and KC425461).

## 3. Results

### (a) Isolation and sequence characterization of TRIM5αs from lagomorphs

**Figure 2.**
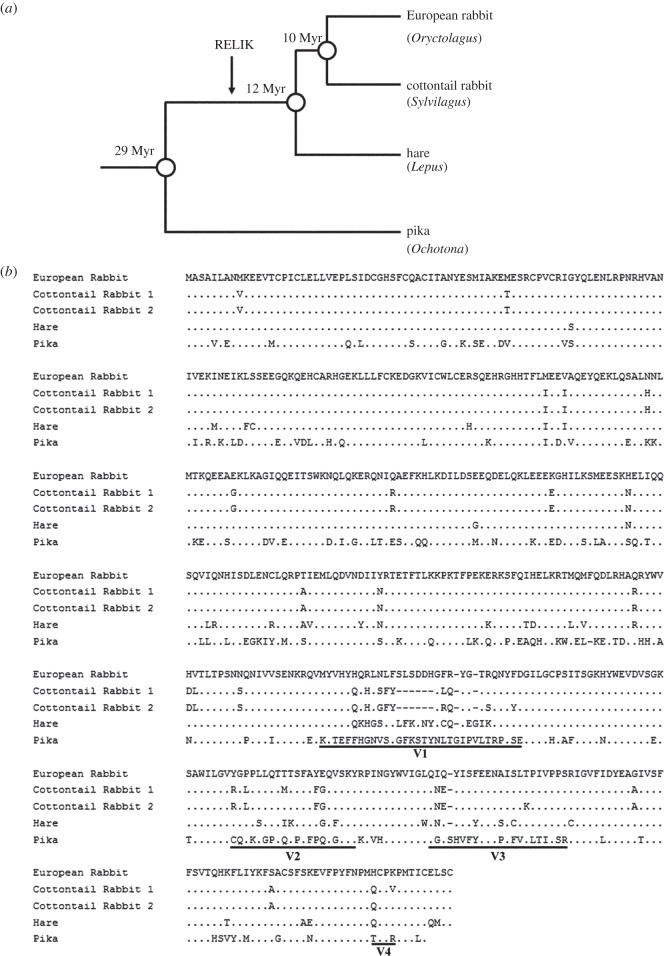
Evolution of TRIM5α in lagomorphs. (*a*) Evolutionary tree of species studied. The time of RELIK acquisition is indicated with an arrow. (*b*) Alignment of the predicted amino acid sequences of TRIM5αs from the European rabbit, cottontail rabbit, hare and pika. The protein sequences were aligned using the ClustalW method in the MegAlign program from DNAstar. Residues identical to the European rabbit sequence are represented by dots, whereas gaps are indicated by dashes. The variable regions V1, V2, V3 and V4 are underlined and annotated.

To examine the influence of RELIK on TRIM5α evolution, we set out to compare the sequences and functional properties of TRIM5α from lagomorphs with and without endogenized RELIK (figure 2*a*). We prepared functional TRIM5α constructs from European and cottontail rabbit that contain RELIK, and from pika which does not [6,8,34]. The TRIM5α of the European and cottontail rabbits was amplified and cloned by RT-PCR from the SIRC and Sf1ep cell lines, respectively. Two different versions of TRIM5α were cloned from cottontail rabbit, presumably corresponding to two different alleles of TRIM5. A source of pika mRNA was not available to us, so the pika construct was prepared by amplifying exon8, containing the primary specificity determinants for retroviral restriction by TRIM5α [31], from pika genomic DNA and fusing it to the RBCC domain of the European rabbit TRIM5α cDNA.

These four clones were sequenced and compared with one another and other lagomorph TRIM5α sequences deposited in the NCBI and EBI databases. The sequence of the pika exon 8 was identical to that found in the draft pika genome sequence. An alignment of the European and the cottontail rabbit (determined here) with the hare [34], and the pika (inferred from the genomic DNA sequence) TRIM5α proteins is shown in figure 2*b*. The sequences show marked diversity, particularly in the regions that corresponded to the variable regions (V1–4) previously identified in the primate TRIM5αs [30,31], with the greatest differences occurring in the V1 region. In this region, the European rabbit and hare sequences are two residues shorter than the pika, whereas the cottontail rabbit alleles lack an additional six residues. In addition, the V3 region of the pika is one residue longer than those of the other species. Differences in the lengths of these regions are frequently observed in primate TRIM5α [30,40].

A particular feature of genes involved in evolutionary conflicts is a high frequency of non-synonymous nucleotide substitutions. We therefore analysed the sequence data for the presence of positively or negatively selected sites using the DataMonkey webserver. Fourteen positively selected codons were identified (table 2); no negatively selected sites were seen. Thirteen of selected sites mapped within the B30.2 domain with 12 present in the different variable regions, consistent with previous observations on TRIM5α and confirming that lagomorph TRIM5α has been subjected to positive selection during the course of evolution [34,35].

**Table 2.** Positively selected residues in lagomorph Trim5s^a^.

codon position^b^	residues present^c^	Bayes factor^d^
328	Q, H, F	329
329	H, G, R	227
330	L, G, N	703
331	S, G, V, N	1112
334	L, G, S	971
341	C, L, F, R	576
342	Q, R, T	152
344	G, E, P	140
346	K, R, T, S	126
389	I, T, Q, M	1534
394	Y, F, P	117
454	K, T, V	133
477	Q, H, T	164
480	K, V, R	119

^a^Sequences were aligned using ClustalX, and the alignment was used in an analysis for positive selection at http://www.datamonkey.org/ using the REL method. Residues that are predicted to be positively selected are listed.

^b^Codon position based on the sequence of the European rabbit.

^c^Single letter amino acid code.

^d^A Bayes factor value between 10 and 100 provides strong evidence for positive selection while those over 100 are decisive.

### (b) Functional characterization of TRIM5α from lagomorphs

**Figure 3.**
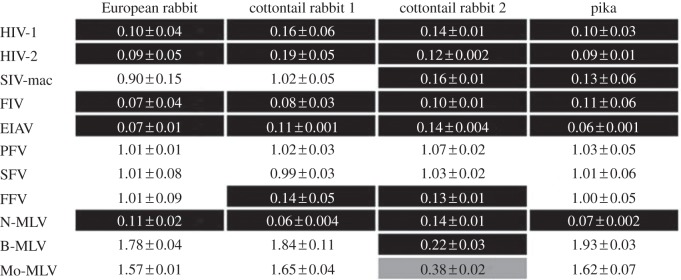
Retrovirus restriction by lagomorph TRIM5αs. CrFK cells were transduced with retroviral vectors carrying the different TRIM5αs as well as the EYFP marker. The transduced cells were challenged 3 days later with a panel of retroviruses carrying the EGFP marker. The ratios of the percentages of infected cells (EGFP positive) containing restriction factor (EYFP positive) to those without restriction factor (EYFP negative) were determined by flow cytometry after a further 2 days. Ratios of less than 0.3 (shaded in black) were taken to represent restriction, whereas ratios greater than 0.7 indicated absence of restriction. Ratios between 0.3 and 0.7 represent partial restriction and are shaded in grey. The numbers represent the average of three experiments.

To examine the anti-retroviral activities of the lagomorph TRIM5αs, CrFK cells were transduced with retroviral vectors encoding the different TRIM5α proteins, and then challenged with a panel of retroviruses, including gammaretroviruses, lentiviruses and foamy viruses (figure 3). Individual TRIM5αs displayed slightly different restriction phenotypes. The foamy viruses PFV and SFV were not restricted by any TRIM5α, whereas the lentiviruses HIV-1, HIV-2, EIAV, FIV and the gammaretrovirus N-MLV were restricted by all four factors. FFV was restricted only by the TRIM5αs from the cottontail rabbit, whereas B-MLV and, to a lesser extent, Mo-MLV were restricted by one of the two cottontail alleles. SIVmac was susceptible to that from the pika and one of the cottontail alleles. Because the pika construct possessed an identical RBCC with the European rabbit, we can conclude that the altered specificity for SIVmac is due to a change in the B30.2 domain. However, changes in the overall profile of restriction activity appear relatively small despite significant changes in TRIM5α sequence.

### (c) Chimeric RELIK lentiviral Gag constructs

**Figure 4.**
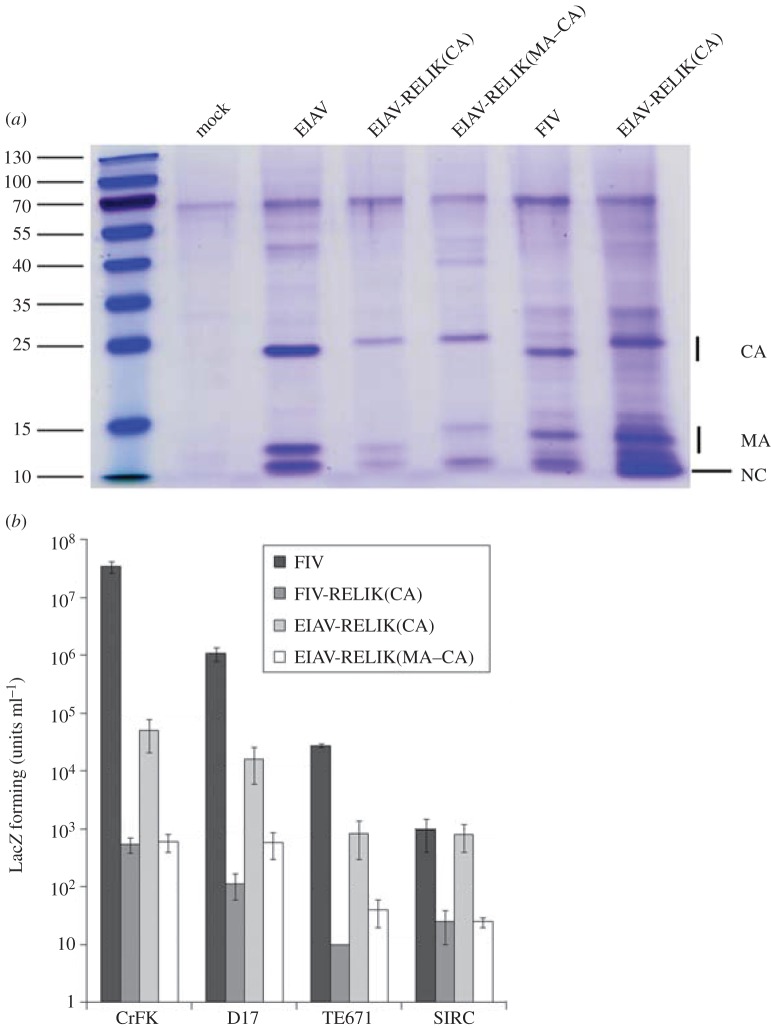
Analysis of chimeric viruses containing the RELIK CA. (*a*) SDS–PAGE analysis of viral supernatants reveals proper processing of CA. 293T cells were transfected with various constructs containing the RELIK CA. Supernatants were harvested 48 h post-transfection and pelleted through a 20% sucrose cushion before separation on a 12% polyacrylamide gel, followed by staining for protein. Sizes of the markers in kDa are indicated on the left, whereas the viral components are indicated on the right. (*b*) Titres of viruses containing RELIK CA in various mammalian cell lines. CrFK (feline), D17 (canine), TE671 (human) or SIRC (European rabbit) cells in 12-well plates were transduced with serial dilutions of viruses and stained for LacZ 48 h post-transduction. The number of blue cells was counted in wells containing between five and 500 blue cells and multiplied by the dilution used. Titres were determined as the number of LacZ forming units per ml of virus. Results shown represent an average of three experiments. (Online version in colour.)

We next wanted to test whether the sequence changes in TRIM5α were associated with an alteration in the ability to restrict RELIK. We had previously described the construction of a chimeric lentiviral Gag, EIAV-RELIK(CA), consisting of the RELIK capsid (CA) in a background of EIAV Gag [7] that produced infectious viruses when co-transfected with an EIAV vector and VSV-G into 293T cells. However, the viral titres were significantly lower than the parental EIAV and could only be measured accurately by endpoint dilution after concentration of the viral supernatant. Before embarking on restriction studies with this virus, we wished to investigate whether we could understand the reasons for this low titre. In one approach, the matrix (MA) of EIAV in the chimeric EIAV-RELIK(CA) Gag was replaced with that from RELIK, resulting in EIAV-RELIK(MA–CA). Alternatively, the CA of FIV was replaced with that from RELIK to test the RELIK CA in the background of FIV Gag, yielding FIV-RELIK(CA). The supernatants from 293T cells transfected with VSV-G, an EIAV vector and either EIAV, EIAV-RELIK(CA) or EIAV-RELIK(MA–CA) Gag-pol were pelleted through 20% sucrose cushions and analysed by PAGE (figure 4*a*). The supernatants from 293T cells transfected with VSV-G, an FIV vector, and either FIV or FIV-RELIK(CA) Gag-pol were analysed in a similar way (figure 4*a*). Bands corresponding to the expected sizes for MA, CA and NC were observed in all viral supernatants compared with the mock control. CA from EIAV-RELIK(CA) and EIAV-RELIK(MA–CA) was larger than that of EIAV, whereas the MA band for EIAV-RELIK(MA–CA) was larger than those from EIAV-RELIK(CA) and EIAV. In addition, the CA band for FIV-RELIK(CA) was larger than that of FIV and the same size as those from EIAV-RELIK(CA) and EIAV-RELIK(MA–CA). These results indicated that properly processed viruses were made from all chimeric Gag-pol constructs. Furthermore, the intensities of the bands from different viruses were comparable, suggesting that the viruses with chimeric Gags were assembled, released and processed with similar efficiencies to the parental Gags.

The infectivities of the viruses with chimeric Gags were investigated by titration on a panel of cells of different origins, including human (TE671), canine (D17), feline (CrFK) and rabbit (SIRC) (figure 4*b*). In general, the titres were highest on feline CrFK cells and lowest in rabbit SIRC cells. This was consistent with previous reports of the existence of several blocks to lenti- and retroviral infection in rabbit cells [41]. Hence, CrFK cells were used for all subsequent studies. Introduction of the RELIK MA into the background of EIAV Gag yielding EIAV-RELIK(MA–CA) resulted in a greater than 10-fold decrease in infectivity compared with the chimeric Gag containing only the RELIK CA (EIAV-RELIK(CA)) in all cell lines. This suggested that the RELIK MA had a detrimental effect on the infectivity of the chimeric viruses. Alternatively, EIAV Gag could not tolerate the large substitution including both MA and CA. Replacing the CA of FIV with that from RELIK resulted in a large decrease in infectivities in all cell lines. These results showed that the Gag containing RELIK CA that yielded the highest titres was EIAV-RELIK(CA). Hence, this was used in all subsequent experiments.

### (d) Restriction of RELIK by TRIM5α from various primates

**Figure 5.**
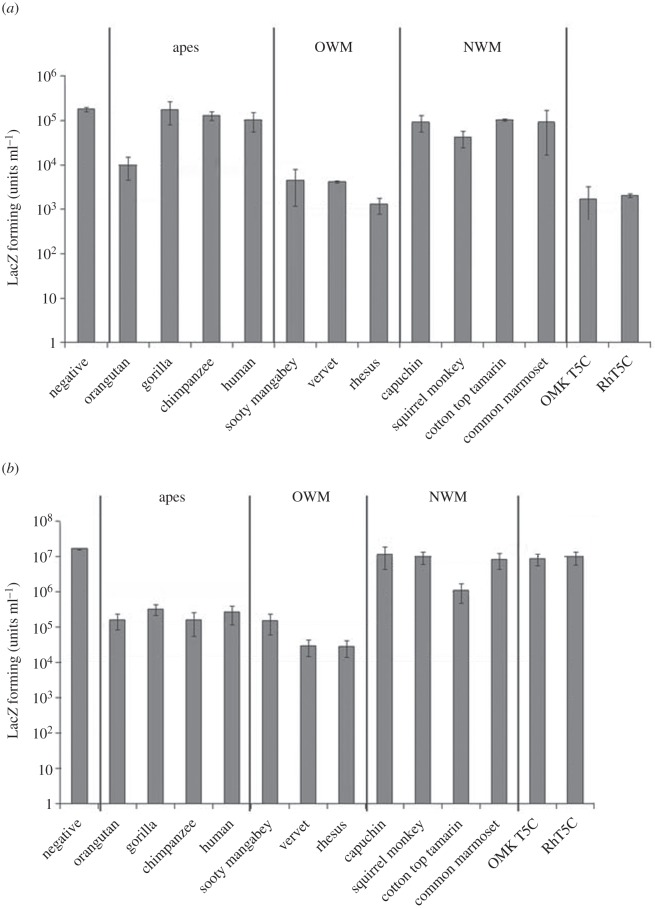
Restriction of EIAV and EIAV-RELIK(CA) by a panel of primate TRIM5αs. CrFK cells were transduced with retroviral vectors carrying TRIM5α with the B30.2 domains from the indicated primates before challenging with serial dilutions of (*a*) EIAV-RELIK(CA) or (*b*) EIAV. The transduced cells were stained for LacZ 48 h later. The number of blue cells was counted in wells containing between five and 500 blue cells and multiplied by the dilution used. Titres were determined as the number of LacZ forming units per ml of virus. The results are an average of three experiments.

To examine whether RELIK was susceptible to TRIM5α restriction, we challenged a panel of permissive CrFK cells transduced with the TRIM5α from different primates with the EIAV-RELIK(CA) virus and EIAV. The results are shown in figure 5. As observed previously, EIAV-RELIK(CA) but not EIAV was restricted by the TRIM5CypA from the owl monkey and rhesus macaque [7]. Unlike EIAV, which is restricted more than 10-fold by the four ape TRIM5αs (orangutan, gorilla, chimpanzee and human), EIAV-RELIK(CA) was restricted only by the orangutan TRIM5α. Both EIAV and EIAV-RELIK(CA) were restricted by the three TRIM5αs from Old World monkeys (sooty mangabey, vervet and rhesus macaque), but not by those from the New World monkeys capuchin, squirrel monkey and common marmoset. EIAV-RELIK(CA) is also resistant to the TRIM5α from the cotton top tamarin (New World monkey), whereas EIAV is modestly restricted (about 10-fold). These results indicated that RELIK can be a target for CA-dependent restriction factors such as TRIM5α. They also show that the RELIK CA is sufficiently different from that of EIAV to enable it to escape from restriction by the TRIM5αs from apes and the cotton top tamarin.

### (e) Restriction of RELIK by lagomorph TRIM5αs

**Figure 6.**
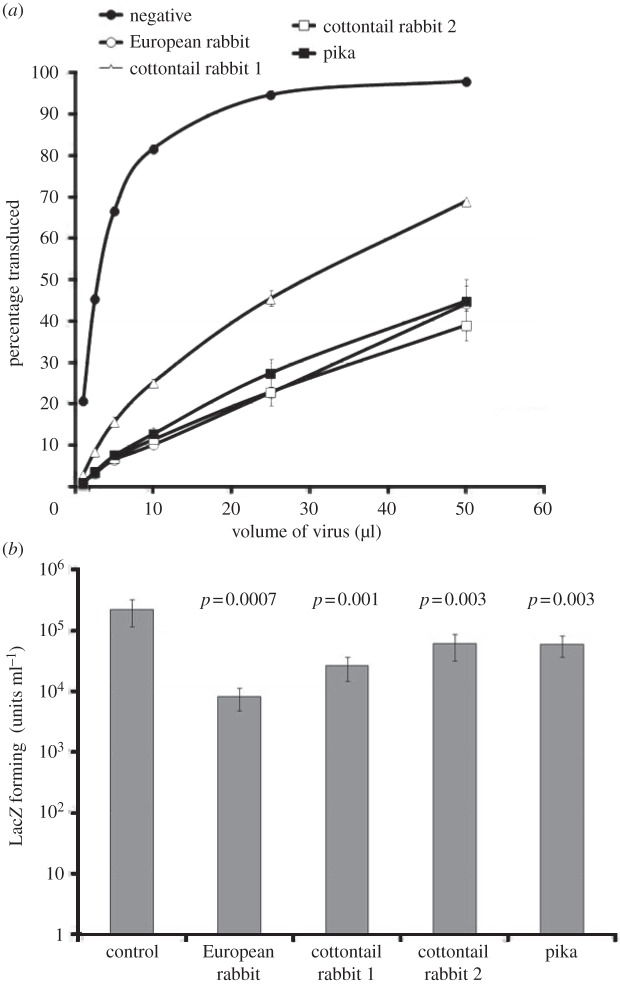
Restriction of EIAV-RELIK(CA) by TRIM5αs from lagomorphs. (*a*) Single cell clones of CrFK cells established after transduction with retroviral vectors carrying TRIM5α from the European rabbit, cottontail rabbit or the chimaera containing the B30.2 domain from the pika were treated with increasing volumes (1, 2.5, 5, 10, 25 and 50 μl) of HIV-1 vector containing the EGFP marker, and the levels of transduction determined by flow cytometry 48 h later. Each data point is an average of two experiments. (*b*) The single cell clones were challenged with serial dilutions of EIAV-RELIK(CA). The transduced cells were stained for LacZ 48 h later. The number of blue cells was counted in wells containing between five and 500 blue cells and multiplied by the dilution factor. Titres were determined as the number of LacZ forming units per ml of virus. The results are an average of three experiments. The *p-*values of Student's *t*-tests between the titres in the control and single cell clones are indicated above the bars.

The results from the sequence studies provided evidence for the presence of selection forces acting on lagomorph TRIM5αs, most likely from retroviruses (figure 3 and [15,34]). Because the lagomorph TRIM5αs were active against various lentiviruses, it was tempting to speculate that RELIK could be involved in driving the evolution of TRIM5α in lagomorphs. To test this hypothesis, single cell clones were derived from CrFK cells transduced with the lagomorph TRIM5α expression constructs by endpoint titration of retroviral vectors carrying the TRIM5α genes on CrFK cells, followed by G418-selection so that single colonies, which were well separated, could be isolated. This was to ensure that the clones contained a single copy of the lagomorph TRIM5α gene. Titration of HIV-1 on these clones revealed very similar levels of restriction by TRIM5 (figure 6*a*) arguing for similar levels of restriction factor expression. The titres of EIAV-RELIK(CA) on each clone were then determined by endpoint dilution and compared with the parental CrFK cell line (figure 6*b*). Compared with the non-transduced control, CrFK cells containing the TRIM5α from the European rabbit were about 30-fold (*p* = 0.0007) less susceptible to EIAV-RELIK(CA), whereas the cells containing TRIM5αs from the cottontail rabbit and pika showed lower levels of restriction, about ninefold (*p* = 0.001) or fourfold (*p* = 0.003) and fourfold (*p* = 0.003), respectively. These results are consistent with the hypothesis that acquisition of RELIK had provided a selective force leading to the evolution of TRIM5α with enhanced restrictive activity directed against RELIK. However, we are unable to discount the possibility of other infectious agent(s) exerting the selective pressure on TRIM5α, resulting in a form which restricted RELIK.

## 4. Discussion

Consideration of the interaction between the various TRIM5αs and their targets reveals three riddles that need answering to claim a full understanding of restriction specificity. First, how can Trim5α from one species recognize and interact with the CA protein of retroviruses from different genera if they share little or no sequence identity? Second, why will Trim5α from multiple species recognize a given virus even though the interacting residues, thought to lie in the variable regions of the Trim5α [30,31], differ significantly? Third, given the wide range of possible interactions reflected by questions one and two, why can a single amino acid change, for example the R332P alteration in human TRIM5α which allows restriction of HIV-1 [42] or the R110E change in CA that controls N/B tropism in MLV [43], have such a major effect on restriction?

The properties of the lagomorph Trim5αs we have examined provide further illustrations of these facets of TRIM5α restriction. They restrict a variety of viruses including lentiviruses, gammaviruses and foamy viruses (figure 3) with very different CA sequences. Pika and European rabbit recognize many of the same viruses despite having very divergent V1, V2 and V3 regions (figure 2). We know that single amino acid changes in V1 can alter the restriction properties of human TRIM5α [42,44,45] but a comparison of the Pika (32 amino acids) and European rabbit (30 amino acids) V1 region shows only three amino acid identities, yet both can restrict HIV-1, HIV2, FIV, EIAV and N-MLV but not PFV. SFV or B-MLV. Interestingly, the two alleles of cottontail rabbit, that are quite closely related (6/64 amino acid differences in V1 plus V2 plus V3), show significant differences in their restriction profiles. It particular, allele-2 was capable of restricting B-MLV, a property of naturally occurring Trim5 alleles not previously observed [31]. We note that multiple alleles of TRIM5α have previously been reported in European brown hare, Iberian hare and European rabbit [35]. It is therefore very tempting to suggest that this represents a further example of balancing selection among TRIM5α alleles [46].

It seems likely that the common fold of the CA protein from the Orthoretrovirinae [47] and similar properties underlying assembly in mature cores [29,47–49], coupled with relatively non-specific interactions between the surfaces of CA and Trim5 [50] will prove important elements in addressing these questions. However, the case of the foamy viruses, which are susceptible to Trim5α restriction (figure 3 and [24]) but seem to form a rather different structure [51], provides an important complicating factor. Determining the structures of co-crystals between TRIM5α and a viral target would go a long way to resolving these issues.

TRIM5α sequences, and therefore their target specificities, have been moulded in large part by a series of positive selection events [32,33,52]. However, the nature of the viruses driving these changes has been the subject of some debate. It is not clear whether the primary driving force for selection is the need to control ERVs, in which case viruses might be present in the species in which selection took place, or driven by exogenous infection in which case the virus might no longer exist or still be present in the species acting as a source for infection. One case in which a specific group of endogenous viruses has been implicated is the PtERV1 family that is present in chimpanzees but not in humans [53]. It was argued that the human TRIM5 changed in response to PtERV exposure protecting humans but leaving them susceptible to a later HIV-1 infection. However, others have subsequently questioned this suggestion [54].

The observations that (i) RELIK is present in some lagomorphs but not others [8,34], (ii) positive selection of TRIM5α was taking place in these animals [34,35], and (iii) our ability to generate an infectious viral vector containing RELIK CA [7] suggested that it should be possible to examine whether the acquisition of RELIK was associated with specific changes in TRIM5α resulting in RELIK restriction. Before embarking on these experiments, we wanted to see whether it was possible to generate a virus with higher titre than our original construct. We found that EIAV tolerates substitution with the RELIK CA better than FIV, despite RELIK sharing the same late domain with FIV, which is different from that in EIAV [55]. This could reflect the closer relatedness between EIAV and RELIK as observed from phylogenetic analyses [6,56]. The substitution seemed to compromise infectivity during early events, because processed Gag in the chimeric viruses was produced at levels similar to parental ones (figure 4). In addition, inclusion of the RELIK MA together with CA in the context of the EIAV Gag further reduced the infectivity without affecting viral production. These results suggest that the different viral components continue to have an impact on each other even after virion maturation. In the absence of any evidence of direct interactions between the cleaved viral products, a possible explanation is that they recruit different cellular components that interact with the other viral products. These cellular factors will be more compatible to homologous viral proteins than those from a less related virus. Examination of these questions might be expected to shed further light on possible roles for Gag products in early, post-entry, events during infection.

Comparison of replication of viral vectors containing the RELIK CA in the presence of TRIM5α from various lagomorphs revealed a gradient of restriction activity increasing from fourfold for pika, through four- and ninefold for the two cottontail alleles and 30-fold for the European rabbit (figure 6*b*). The various TRIM5α proteins show very similar effects on HIV-1 (figures 2 and 6*a*) arguing against any possible effects of TRIM5α expression level in the different clones. Such an increase in restriction of RELIK is consistent with a process driven by RELIK exposure. However, the increase is relatively small and these data by themselves do not address the question of whether selection is mediated by endogenous or exogenous viruses.

In the light of the absence of RELIK in the pika genome, one explanation for the low level of restriction of RELIK by the pika TRIM5α could be that the ancestors of the pika had not been exposed to the virus and, hence, the pika TRIM5α was not selected for providing resistance to this virus. Alternatively, the low level of restriction could be vestige of a successful protection against RELIK: the pika TRIM5α could have evolved under selection pressure from other retroviruses following the extinction of RELIK.

We also note that restriction is strongest by the TRIM5α from the European rabbit, which is the source of the RELIK sequence used in this study, possibly suggesting that the endogenized virus is most susceptible to the TRIM5α from its host. One target of TRIM5α restriction in HIV-1 has been mapped to the cyclophilin A-binding loop between alpha-helices 4 and 5 of the CA [57,58], though other regions may be important [59]. An exchange of the helix 4/5 region between SIV_mac_ and HIV-2 resulted in altered susceptibilities to restriction by the Rhesus TRIM5α [60]. Although the RELIK sequences seem to be well conserved between the different genera in Leporidae with more than 90% homology, comparison of the cyclophilin A-binding loop between the RELIK CA from the European rabbit and the one sequence of the orthologue found in hares (accession no. FJ493032) revealed four substitutions and two deletions. Because small changes in this region are sufficient to influence restriction, it is tempting to speculate that the RELIK from hares will have different susceptibilities to lagomorph TRIM5αs. To understand more about the relationship between endogenized lentiviruses and TRIM5α, it will be necessary to investigate the restriction of RELIK orthologues found in other genera of the Leporidae by the range of lagomorph TRIM5αs.

## References

[1] Stoye JP 2012 Studies of endogenous retroviruses reveal a continuing evolutionary saga. Nat. Rev. Microbiol. 10, 395–406 (doi:10.1038/nrmicro2783) 22565131 10.1038/nrmicro2783

[2] Feschotte C Gilbert C 2012 Endogenous viruses: insights into viral evolution and impact on host biology. Nat. Rev. Genet. 13, 283–296 (doi:10.1038/nrg3199) 22421730 10.1038/nrg3199

[3] Goodchild NL Wilkinson DA Mager DL 1993 Recent evolutionary expansion of a subfamily of RTVL-H human endogenous retrovirus-like elements. Virology 196, 778–788 (doi:10.1006/viro.1993.1535) 8372448 10.1006/viro.1993.1535

[4] Emerman M Malik HS 2010 Paleovirology: modern consequences of ancient viruses. PLoS Biol. 8, e1000301 (doi:10.1371/journal.pbio.1000301) 20161719 10.1371/journal.pbio.1000301 PMC2817711

[5] Patel MR Emerman M Malik HS 2011 Paleovirology: ghosts and gifts of viruses past. Curr. Opin. Virol. 1, 304–309 (doi:10.1016/j.coviro.2011.06.007) 22003379 10.1016/j.coviro.2011.06.007 PMC3190193

[6] Katzourakis A Tristem M Pybus OG Gifford RJ 2007 Discovery and analysis of the first endogenous lentivirus. Proc. Natl Acad. Sci. USA 104, 6261–6265 (doi:10.1073/pnas.0700471104) 17384150 10.1073/pnas.0700471104 PMC1851024

[7] Goldstone DC Yap MW Robertson LE Haire LF Taylor WR Katzourakis A Stoye JP Taylor IA 2010 Structural and functional analysis of prehistoric lentiviruses uncovers an ancient molecular interface. Cell Host Microbe 8, 248–259 (doi:10.1016/j.chom.2010.08.006) 20833376 10.1016/j.chom.2010.08.006

[8] Keckesova Z Ylinen LM Towers GJ Gifford RJ Katzourakis A 2009 Identification of a RELIK orthologue in the European hare (*Lepus europaeus*) reveals a minimum age of 12 million years for the lagomorph lentiviruses. Virology 384, 7–11 (doi:10.1016/j.virol.2008.10.045) 19070882 10.1016/j.virol.2008.10.045 PMC3556577

[9] van der Loo W Abrantes J Esteves PJ 2009 Sharing of endogenous lentiviral gene fragments among leporid lineages separated for more than 12 million years. J. Virol. 83, 2386–2388 (doi:10.1128/JVI.01116-08) 19109386 10.1128/JVI.01116-08 PMC2643718

[10] Malim MH Bieniasz PD 2012 HIV restriction factors and mechanisms of evasion. Cold Spring Harb. Perspect. Med. 2, a006940 (doi:10.1101/cshperspect.a006940) 22553496 10.1101/cshperspect.a006940 PMC3331687

[11] Best S Le Tissier P Towers G Stoye JP 1996 Positional cloning of the mouse retrovirus restriction gene *Fv1*. Nature 382, 826–829 (doi:10.1038/382826a0) 8752279 10.1038/382826a0

[12] Stremlau M Owens CM Perron MJ Kiessling M Autissler P Sodroski J 2004 The cytoplasmic body component TRIM5a restricts HIV-1 infection in Old World monkeys. Nature 427, 848–853 (doi:10.1038/nature02343) 14985764 10.1038/nature02343

[13] Ylinen LMJ Keckesova Z Webb BLJ Gifford RJM Smith TPL Towers GJ 2006 Isolation of an active Lv1 gene from cattle indicates that tripartite motif protein-mediated innate immunity to retroviral infection is widespread among mammals. J. Virol. 80, 7332–7338 (doi:10.1128/JVI.00516-06) 16840314 10.1128/JVI.00516-06 PMC1563707

[14] Si Z 2006 Evolution of a cytoplasmic tripartite motif (TRIM) protein in cows that restricts retroviral infection. Proc. Natl Acad. Sci. USA 103, 7454–7459 (doi:10.1073/pnas.0600771103) 16648259 10.1073/pnas.0600771103 PMC1464360

[15] Schaller T Hué S Towers GJ 2007 An active TRIM5 protein in rabbits indicates a common antiviral ancestor for mammalian TRIM5 proteins. J. Virol. 81, 11 713–11 721 (doi:10.1128/JVI.01468-07) 10.1128/JVI.01468-07 PMC2168759 17728224

[16] Boudinot P van der Aa LM Jouneau L Du Pasquier L Pontarotti P Briolat V Benmansour A Levraud J-P 2011 Origin and evolution of TRIM proteins: new insights from the complete TRIM repertoire of zebrafish and pufferfish. PLoS ONE 6, e22022 (doi:10.1371/journal.pone.0022022) 21789205 10.1371/journal.pone.0022022 PMC3137616

[17] Nisole S Stoye JP Saïb A 2005 Trim family proteins: retroviral restriction and antiviral defence. Nat. Rev. Microbiol. 3, 799–808 (doi:10.1038/nrmicro1248) 16175175 10.1038/nrmicro1248

[18] Han K Lou DI Sawyer SL 2011 Identification of a genomic reservoir for new TRIM genes in primate genomes. PLoS Genet. 7, e1002388 (doi:10.1371/journal.pgen.1002388) 22144910 10.1371/journal.pgen.1002388 PMC3228819

[19] Short KM Cox TC 2006 Sub-classification of the RBCC/TRIM superfamily reveals a novel motif necessary for microtubule binding. J. Biol. Chem. 281, 8970–8980 (doi:10.1074/jbc.M512755200) 16434393 10.1074/jbc.M512755200

[20] Sardiello M Cairo S Fontanella B Ballabio A Meroni G 2008 Genomic analysis of the TRIM family reveals two groups of genes with distinct evolutionary properties. BMC Evol. Biol. 8, 225 (doi:10.1186/1471-2148-8-225) 18673550 10.1186/1471-2148-8-225 PMC2533329

[21] Keckesova Z Ylinen LMJ Towers GJ 2004 The human and African green monkey TRIM5*α* genes encode Ref1 and Lv1 retroviral restriction factor activities. Proc. Natl Acad. Sci. USA 101, 10 780–10 785 (doi:10.1073/pnas.0402474101) 10.1073/pnas.0402474101 PMC490011 15249687

[22] Hatziioannou T Perez-Caballero D Yang A Cowan S Bieniasz PD 2004 Retrovirus resistance factors Ref1 and Lv1 are species-specific variants of TRIM5*α*. Proc. Natl Acad. Sci. USA 101, 10 774–10 779 (doi:10.1073/pnas.0402361101) 10.1073/pnas.0402361101 PMC490010 15249685

[23] Yap MW Nisole S Lynch C Stoye JP 2004 Trim5*α* protein restricts both HIV-1 and murine leukemia virus. Proc. Natl Acad. Sci. USA 101, 10 786–10 791 (doi:10.1073/pnas.0402876101) 10.1073/pnas.0402876101 PMC490012 15249690

[24] Yap MW Lindemann D Stanke N Reh J Westphal D Hanenberg H Ohkural S Stoye JP 2008 Restriction of foamy viruses by primate Trim5alpha. J. Virol. 82, 5429–5439 (doi:10.1128/JVI.02462-07) 18367529 10.1128/JVI.02462-07 PMC2395188

[25] Diehl WE Stansell E Kaiser SM Emerman M Hunter E 2008 Identification of post-entry restrictions to Mason–Pfizer monkey virus infection in New World monkey cells. J. Virol. 82, 11 140–11 151 (doi:10.1128/JVI.00269-08) 10.1128/JVI.00269-08 PMC2573280 18799582

[26] Stremlau M 2006 Specific recognition and accelerated uncoating of retroviral capsids by the TRIM5*α* restriction factor. Proc. Natl Acad. Sci. USA 103, 5514–5519 (doi:10.1073/pnas.0509996103) 16540544 10.1073/pnas.0509996103 PMC1459386

[27] Yap MW Mortuza GB Taylor IA Stoye JP 2007 The design of artificial retroviral restriction factors. Virology 365, 302–314 (doi:10.1016/j.virol.2007.04.005) 17493656 10.1016/j.virol.2007.04.005

[28] Diaz-Griffero F Qin X-R Hayashi F Kigawa T Finzi A Sarnak Z Lienlaf M Yokoyama S Sodroski J 2009 A B-box 2 surface patch important for TRIM5*α* self-association, capsid binding avidity and retrovirus restriction. J. Virol. 83, 10 737–10 751 (doi:10.1128/JVI.01307-09) 10.1128/JVI.01307-09 PMC2753111 19656869

[29] Ganser-Pornillos BK Chandrasekaran V Pornillos O Sodroski JG Sundquist WI Yeager M 2011 Hexagonal assembly of a restricting TRIM5*α* protein. Proc. Natl Acad. Sci. USA 108, 534–539 (doi:10.1073/pnas.1013426108) 21187419 10.1073/pnas.1013426108 PMC3021009

[30] Song B Gold B O'hUigin C Javanbakht M Li X Stremlau M Winkler C Dean M Sodroski J 2005 The B30.2(SPRY) domain of retroviral restriction factor TRIM5*α* exhibits lineage-specific length and sequence variation in primates. J. Virol. 79, 6111–6121 (doi:10.1128/JVI.79.10.6111-6121.2005) 15857996 10.1128/JVI.79.10.6111-6121.2005 PMC1091705

[31] Ohkura S Yap MW Sheldon T Stoye JP 2006 All three variable regions of the TRIM5α B30.2 domain can contribute to the specificity of retrovirus restriction. J. Virol. 80, 8554–8565 (doi:10.1128/JVI.00688-06) 16912305 10.1128/JVI.00688-06 PMC1563890

[32] Sawyer SL Wu LI Emerman M Malik HS 2005 Positive selection of primate TRIM5*α* identifies a critical species-specific retroviral restriction domain. Proc. Natl Acad. Sci. USA 102, 2832–2837 (doi:10.1073/pnas.0409853102) 15689398 10.1073/pnas.0409853102 PMC549489

[33] Johnson WE Sawyer SL 2009 Molecular evolution of the antiretroviral *TRIM5* gene. Immunogenetics 61, 163–178 (doi:10.1007/s00251-009-0358-y) 19238338 10.1007/s00251-009-0358-y

[34] Fletcher AJ Hue S Schaller T Pillay D Towers GJ 2010 Hare TRIM5α restricts divergent retroviruses and exhibits significant sequence variation from closely related lagomorpha TRIM5 genes. J. Virol. 84, 12 463–12 468 (doi:10.1128/JVI.01514-10) 10.1128/JVI.01514-10 PMC2976397 20861252

[35] de Matos AL van der Loo W Areal H Lanning DK Esteves PJ 2011 Study of sylvilagus rabbit TRIM5alpha species-specific domain: how ancient endoviruses could have shaped the antiviral repertoire in Lagomorpha. BMC Evol. Biol. 11, 294 (doi:10.1186/1471-2148-11-294) 21982459 10.1186/1471-2148-11-294 PMC3208668

[36] Loewen N Barraza R Whitwam T Saenz DT Kemler I Poeschla E 2003 FUV vectors. Methods Mol. Biol. 229, 251–271 12824636 10.1385/1-59259-393-3:251

[37] Mitrophanous K Yoon S Rohll J Patil D Wilkes F Kim V Kingsman S Kingsman A Mazaratis N 1999 Stable gene transfer to the nervous system using a non-primate lentiviral vector. Gene Ther. 6, 1808–1818 (doi:10.1038/sj.gt.3301023) 10602376 10.1038/sj.gt.3301023

[38] Griffin SD Allen JF Lever AM 2001 The major human immunodeficiency virus type 2 (HIV-2) packaging signal is present on all HIV-2 RNA species: cotranslational RNA encapsidation and limitation of Gag protein confer specificity. J. Virol. 75, 12 058–12 069 (doi:10.1128/JVI.75.24.12058-12069.2001) 10.1128/JVI.75.24.12058-12069.2001 PMC116101 11711596

[39] Soneoka Y Cannon PM Ramsdale EE Griffiths JC Romano G Kingsman SM Kingsman AJ 1995 A transient three-plasmid expression system for the production of high titer retroviral vectors. Nucleic Acids Res. 23, 628–633 (doi:10.1093/nar/23.4.628) 7899083 10.1093/nar/23.4.628 PMC306730

[40] Rahm N 2011 Unique spectrum of activity of prosimian TRIM5*α* against exogenous and endogenous retroviruses. J. Virol. 85, 4173–4183 (doi:10.1128/JVI.00075-11) 21345948 10.1128/JVI.00075-11 PMC3126249

[41] Cutino-Moguel T Fassati A 2006 A phenotypic recessive, post-entry block in rabbit cells that results in aberrant trafficking of HIV-1. Traffic 7, 978–992 (doi:10.1111/j.1600-0854.2006.00449.x) 16882040 10.1111/j.1600-0854.2006.00449.x PMC1934423

[42] Yap MW Nisole S Stoye JP 2005 A single amino acid change in the SPRY domain of human Trim5*α* leads to HIV-1 restriction. Curr. Biol. 15, 73–78 (doi:10.1016/j.cub.2004.12.042) 15649369 10.1016/j.cub.2004.12.042

[43] Kozak CA Chakraborti A 1996 Single amino acid changes in the murine leukemia virus capsid protein gene define the target for *Fv1* resistance. Virology 225, 300–306 (doi:10.1006/viro.1996.0604) 8918916 10.1006/viro.1996.0604

[44] Li Y Li X Stremlau M Lee M Sodroski J 2006 Removal of arginine 332 allows human TRIM5*α* to bind human immunodeficiency virus capsids and to restrict infection. J. Virol. 80, 6738–6744 (doi:10.1128/JVI.00270-06) 16809279 10.1128/JVI.00270-06 PMC1489046

[45] Maillard PV Reynard S Serhan F Turelli P Trono D 2007 Interfering residues narrow the spectrum of MLV restriction by human TRIM5alpha. PLoS Pathog. 3, e200 (doi:10.1371/journal.ppat.0030200) 18166079 10.1371/journal.ppat.0030200 PMC2156100

[46] Newman RM 2006 Balancing selection and the evolution of functional polymorphism in Old World monkey TRIM5*α*. Proc. Natl Acad. Sci. USA 103, 19 134–19 139 (doi:10.1073/pnas.0605838103) 10.1073/pnas.0605838103 PMC1679755 17142324

[47] Mortuza GB Goldstone DC Pashley C Haire LF Palmarini M Taylor WR Stoye JP Taylor IA 2009 Structure of the capsid amino-terminal domain from the betaretrovirus, Jaagsiekte sheep retrovirus. J. Mol. Biol. 386, 1179–1192 (doi:10.1016/j.jmb.2008.10.066) 19007792 10.1016/j.jmb.2008.10.066

[48] Li S Hill CP Sundquist WI Finch JT 2000 Image reconstruction of helical assemblies of the HIV-1 CA protein. Nature 407, 409–413 (doi:10.1038/35030177) 11014200 10.1038/35030177

[49] Mortuza GB Haire LF Stevens A Smerdon SJ Stoye JP Taylor IA 2004 High-resolution structure of a retroviral capsid hexameric amino-terminal domain. Nature 431, 481–485 (doi:10.1038/nature02915) 15386017 10.1038/nature02915

[50] Ohkura S Goldstone DC Yap MW Holden-Dye K Taylor IA Stoye JP 2011 Novel escape mutants suggest an extensive TRIM5alpha binding site spanning the entire outer surface of the murine leukemia virus capsid protein. PLoS Pathog. 7, e1002011 (doi:10.1371/journal.ppat.1002011) 21483490 10.1371/journal.ppat.1002011 PMC3068999

[51] Goldstone DC 2013 A unique spumavirus Gag N-terminal domain with functional properties of orthoretroviral Matrix and Capsid. PLoS Pathog. 9, e1003376 (doi:10.1371/journal.ppat.1003376) 23675305 10.1371/journal.ppat.1003376 PMC3649970

[52] Meyerson NR Sawyer SL 2011 Two-stepping through time: mammals and viruses. Trends Microbiol. 19, 286–294 (doi:10.1016/j.tim.2011.03.006) 21531564 10.1016/j.tim.2011.03.006 PMC3567447

[53] Kaiser SM Malik HS Emerman M 2007 Restriction of an extinct retrovirus by the human TRIM5*α* antiviral protein. Science 316, 1756–1758 (doi:10.1126/science.1140579) 17588933 10.1126/science.1140579

[54] Perez-Caballero D Soll SJ Bieniasz PD 2008 Evidence for restriction of ancient primate gammaretroviruses by APOBEC3 but not TRIM5*α* proteins. PLoS Pathog. 4, e1000181 (doi:10.1371/journal.ppat.1000181) 18927623 10.1371/journal.ppat.1000181 PMC2564838

[55] Luttge BG Shehu-Xhilaga M Demirov DG Adamson CS Soheilian F Nagashima K Stephen AG Fisher RJ Freed EO 2008 Molecular characterization of feline immunodeficiency virus budding. J. Virol. 82, 2106–2119 (doi:10.1128/JVI.02337-07) 18094166 10.1128/JVI.02337-07 PMC2258934

[56] Gifford RJ Katzourakis A Tristem M Pybus OG Winters M Shafer RW 2008 A transitional endogenous lentivirus from the genome of a basal primate and implications for lentivirus evolution. Proc. Natl Acad. Sci. USA 105, 20 362–20 367 (doi:10.1073/pnas.0807873105) 19075221 10.1073/pnas.0807873105 PMC2603253

[57] Münk C Brandt SM Lucero G Landau NR 2002 A dominant block to HIV-1 replication at reverse transcription in simian cells. Proc. Natl Acad. Sci. USA 99, 13 843–13 848 (doi:10.1073/pnas.212400099) 12368468 10.1073/pnas.212400099 PMC129785

[58] Hatziioannou T Cowan S von Schwedler UK Sundquist WI Bieniasz PD 2004 Species-specific tropism determinants in the human immunodeficiency virus type 1 capsid. J. Virol. 78, 6005–6012 (doi:10.1128/JVI.78.11.6005-6012.2004) 15140998 10.1128/JVI.78.11.6005-6012.2004 PMC415825

[59] Kono K Song H Yokoyama M Sato H Shioda T Nakayama EE 2010 Multiple sites in the N-terminal half of simian immunodeficiency virus capsid protein contribute to evasion from rhesus monkey TRIM5alpha-mediated restriction. Retrovirology 7, 72 (doi:10.1186/1742-4690-7-72) 20825647 10.1186/1742-4690-7-72 PMC2944288

[60] Ylinen LMJ Keckesova Z Wilson SJ Ranasinghe S Towers GJ 2005 Differential restriction of human immunodeficiency virus type 2 and simian immunodeficiency virus SIVmac by TRIM5*α* alleles. J. Virol. 79, 11 580–11 587 (doi:10.1128/JVI.79.18.11580-11587.2005) 10.1128/JVI.79.18.11580-11587.2005 PMC1212619 16140735

